# Optical coherence tomography needle probe for real-time visualization of temperature-induced phase changes within subcutaneous fatty tissue

**DOI:** 10.1117/1.JBO.30.3.035002

**Published:** 2025-03-11

**Authors:** Hinnerk Schulz-Hildebrandt, Michael Wang-Evers, Naja Meyer-Schell, Daniel Karasik, Malte J. Casper, Tim Eixmann, Felix Hilge, Reginald Birngruber, Dieter Manstein, Gereon Hüttmann

**Affiliations:** a Universität zu Lübeck, Institute of Biomedical Optics, Lübeck, Germany; b Airway Research Center North (ARCN), Member of the German Center of Lung Research (DZL), Gießen, Germany; c Medical Laser Center Lübeck, Lübeck, Germany; d Massachusetts General Hospital, Harvard Medical School, Wellman Center for Photomedicine, Boston, Massachusetts, United States; e Massachusetts General Hospital, Harvard Medical School, Cutaneous Biology Research Center, Department of Dermatology, Boston, Massachusetts, United States; f Columbia University, Laboratory for Functional Optical Imaging, Department of Biomedical Engineering, New York, United States

**Keywords:** optical coherence tomography, endoscope, fiber probe, cryolipolysis

## Abstract

**Significance**: Selective cryolipolysis is a widely used aesthetic procedure that cools subcutaneous adipose tissue to temperatures as low as −11°C to induce fat cell destruction. However, real-time monitoring techniques are lacking, limiting the ability to optimize safety and efficacy. Traditional imaging methods either fail to provide adequate penetration depth or lack the resolution necessary for visualizing subcutaneous fatty tissue dynamics.

**Aim**: This paper aims to demonstrate that an optical coherence tomography (OCT) needle probe can be used for real-time observation of temperature-induced changes in subcutaneous fatty tissue, potentially enhancing the assessment and optimization of cryolipolysis procedures.

**Approach**: We developed a side-viewing OCT-based needle probe designed for subcutaneous imaging. The probe consists of a fiber-optic system encased in a transparent, biocompatible polymer catheter with an outer diameter of 900  μm. A 49-degree angled fiber enables imaging, while a piezoelectric scanning system moves the fiber transversely within the catheter. The probe achieves a lateral resolution of <15  μm, a working distance of 600  μm, and a lateral field of view dictated by the scanning system length. OCT imaging was performed on porcine skin with a subcutaneous fat layer >3 cm thick during controlled heating and cooling.

**Results**: OCT imaging revealed increased optical scattering in subcutaneous fatty tissue during cooling, corresponding to the phase transition from liquid to solid. This effect was reversible upon warming, indicating that OCT can dynamically monitor adipocyte crystallization in real time. The observed transition temperatures varied, likely due to differences in lipid composition.

**Conclusions**: OCT-based needle imaging enables direct, high-resolution visualization of adipocyte crystallization, offering a potential tool for optimizing selective cryolipolysis treatments. This technology could improve safety and efficacy by providing real-time feedback on tissue response, facilitating a better understanding of the cooling-induced fat reduction process.

## 1 Introduction

Selective cryolipolysis has been developed as a noninvasive method of reducing subcutaneous fat without damaging the skin.^1^ Since its FDA clearance in 2010, selective cryolipolysis, commercially known as CoolSculpting^®^, has become a popular clinical procedure for noninvasive fat removal. It serves as an alternative to invasive fat removal procedures such as liposuction. Due to its noninvasiveness, there is virtually no patient downtime, and it eliminates many of the risks of surgical interventions, including scarring, infection, necrosis, and even death.^2^ Furthermore, it provides an attractive option for localized removal of fatty tissue. Diet and exercise are generally seen as methods to reduce fatty tissue, but those have their challenges and generally do not allow reducing fatty tissue in localized, targeted areas such as the flanks or the upper arms. Selective cryolipolysis involves controlled and precise localized cooling of adipose fat through the skin. It is believed that the mechanism of fat reduction involves cooling-induced crystallization of the lipids in the adipocytes, which is known to occur above the temperature of water freezing. For a few weeks post procedure, a localized inflammatory response induced by fat crystallization results in apoptosis and necrosis of the adipocytes and, ultimately, a partial loss of fatty tissue. This is largely achieved without damage to the dermis or epidermis, even though cooling is applied to the skin surface. Dermal and epidermal tissues have a higher water content and are less sensitive to cold than adipose tissue.^3^ In addition, a glycerol-based antifreeze is topically applied to the skin to further protect the epidermis from freeze injury. Thus, the inflammatory response is located exclusively in adipose tissue. Although selective cryolipolysis is generally considered a safe treatment, some clinical trials have reported various side effects of the treatment, including erythema, edema, bruising, and transient neuralgia.^4^ Paradoxical adipose hyperplasia, which presents as a delayed, localized increase in fatty tissue volume, has also been presented as a relatively rare adverse effect. Despite^5^^,^^6^ these risks, selective cryolipolysis has become a market success with widespread use in dermatological clinics, with more than 10 million treatments performed at the time of this paper being published. However, the results of treatment can differ from person to person, as various factors, including gender, diet, and hormonal balance, impact the local lipid composition and therefore the crystallization temperature of the fat of the person.^7^^,^^8^ As adipocyte crystallization is considered an important factor for cold-induced adipocyte damage, a method for imaging the formation of crystallization of adipocytes during cryolipolysis can provide important insights and help to improve the treatment conditions (e.g., temperature, duration, skin contact, and applicator geometry) to achieve better outcome. In addition, it will help to improve the biological understanding of cryolipolysis, allowing further exploration of the effects of various diets, repeated treatments, and other factors on the efficacy of the procedure.

Currently, tissue effects of selective cryolipolysis can only be observed by gross examination or posttreatment via histology. It has been shown that optical methods, including near-infrared spectroscopy and optical coherence tomography (OCT), can allow for the observation of temperature-induced changes in subcutaneous adipose tissues.^9^ During fat crystallization, there is a localized increase in optical scattering, which OCT imaging can measure with high spatial resolution.^9^

Real-time imaging with cellular resolution of subcutaneous fatty tissue during the cooling procedure would allow the study of the process of lipid crystallization within adipocytes. This information could be used to correlate the extent of crystallization with subsequent fat volume reduction. In contrast to ultrasound imaging, high-resolution OCT imaging would allow the investigation of the distribution of fat cell size and adipose tissue physiology, which could help to better understand the mechanism of selective cryolipolysis. However, the major limitation of all optical methods, including OCT, is the limited penetration depth in biological tissue.^10^ Therefore, it is not possible to directly image the subcutaneous fat. Endoscopic OCT devices, however, allow for imaging within the human body.^11^^,^^12^ Small-diameter side-viewing needle probes, which were first demonstrated by Li et al.,^13^ can be inserted directly into the tissue and rotated as they are pulled out. The resulting helical OCT scan is then assembled into a 3D volumetric image.^14^ Due to the friction of the probe with the surrounding tissue, a measurement with reproducible localization is not possible after inserting and removing the probe across several consecutive scans. To overcome these limitations, McLaughlin et al. presented a traversing needle probe,^15^^,^^16^ in which the OCT fiber is placed within another stationary stainless-steel needle and is moved back and forth in the axial direction to record a two-dimensional depth image. The imaging is performed through an unsealed window on the outer needle. The use of a protective window was not possible due to the short working distance of the optics.

Here, we demonstrate a catheter traversing needle probe (CTNP) with a 900-μm outer diameter that measures changes in subcutaneous fat in a 10  mm×2.5  mm field of view by OCT. The objective of this study was to place the focal plane at a distance of ∼1.5  mm from the outer surface of the probe and to increase the lateral resolution by compensating for astigmatism. Therefore, the design of the entirely fiber-based optics was optimized by calculating the beam propagation using the split-step beam propagation method (SS-BPM).^17^^,^^18^ Through astigmatism compensation, a lateral resolution of 10  μm was achieved. The concept of the traversing needle probe was extended to a larger lateral field of view (FOV) using a transparent plastic catheter rather than a second stainless steel needle with a window at the side. The CTNP is able to quantify the distribution of adipocyte cell size and to image temperature-induced phase changes of subcutaneous fatty tissue in real time.

## 2 Methods

### 2.1 Manufacturing of the Catheter Traversing Needle Probe

**Fig. 1:**
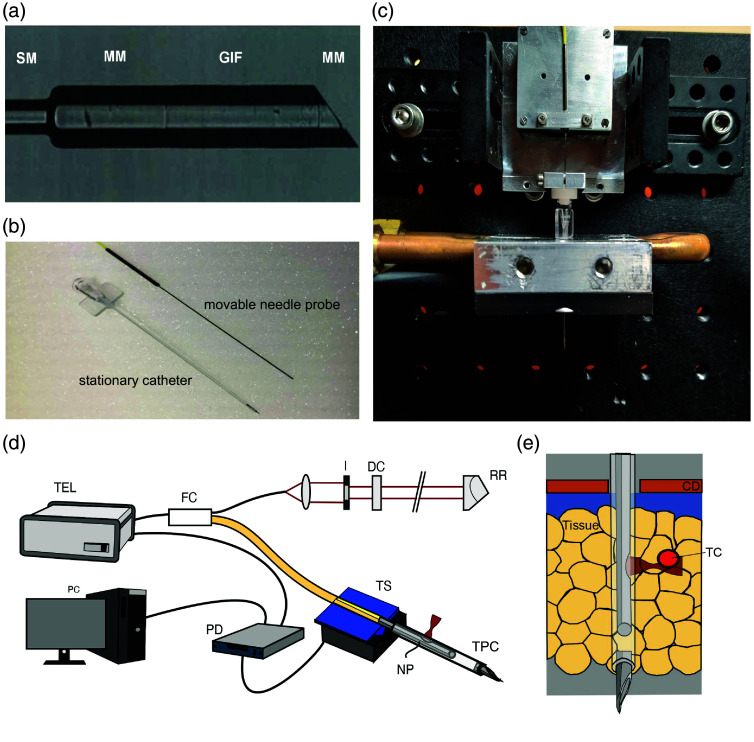
Design of the catheter traversing needle probe (CTNP). (a) The focusing optics were made by splicing four different fibers together: a single-mode fiber (SM), a step-index multimode fiber (MM) to enlarge the beam, a multimode GRIN fiber (GIF) for focusing, and a second piece of angle-polished MM fiber to redirect the beam. (b) Image of the inner movable needle probe (top) and the outer stationary catheter (bottom) equipped with a lancet tip. (c) Fully assembled mechanical setup with the probe, catheter, and scanning stage. (d) Schematic representation of the OCT setup with the needle probe. The Telesto-II OCT system (TEL) was coupled through a fiber coupler (FC) to the reference stage, which contains a collimating lens, an iris diaphragm (I), and a retroreflector (RR). The needle probe (NP) is connected to the second arm of the fiber coupler. Axial scanning of the needle probe in the transparent plastic catheter (TPC) is performed by a linear translation stage (TS), which is driven by a piezo driver (PD). The OCT system and the piezo controller were coupled to the same PC. (e) Experimental setup for measuring the temperature-induced phase change of subcutaneous fat tissue *ex vivo*. The tissue specimen is in contact with the cooling/heating device (CD) and is equipped with a thermocouple (TC), which is placed so that it is visible in the CNTP image.

A crucial element of the CNTP is the focusing optics, which was built by splicing different optical fibers together [Fig. 1(a)]. The starting point for the design was a single-mode fiber (SMF-28-J9, Thorlabs, Newton, New Jersey, United States) that delivers the OCT radiation. To enlarge the beam diameter to around 100  μm, a glass spacer, made from a 220-μm diameter step-index multimode fiber (FG200LEA, Thorlabs, New Jersey, United States) was spliced to the single-mode fiber, and precisely cleaved to a length of 600  μm by a glass processor, GPX 3800 (Thorlabs, Newton, New Jersey, United States). The expanded beam was focused by an 800-μm-long piece of 0.22 NA GRIN multimode fiber (GFW200, Fiberware, Berlin, Germany), which was spliced to the step-index multimode fiber. The beam was deflected sideways by a truncated cylinder made from a second piece of the glass spacer step-index multimode fiber. After splicing to the GRIN fiber, the multimode fiber was cleaved to a length of 240  μm and polished to an angle of 49 deg using a bare fiber polisher (Trig™ Bare Fiber Polisher, Krelltech, Neptune City, New Jersey, United States).

The entirely fiber-based optics was then placed in a customized 55-mm-long stainless-steel tube (Unimed S.A., Lausanne, Switzerland), with an inner diameter of 220  μm and a wall thickness of 85  μm. At the distal end of the tube, an aperture with a diameter of 150  μm was created by electro-erosion to allow the beam to exit laterally. Low-viscosity UV-curing adhesive (NOA 86H, Norland Products Inc., Cranbury, United States) was used to form the exit window. The adhesive was applied several times in small quantities, allowing for time to cure between applications. This ensured that the adhesive would not run down the back of the angled surface of the fiber tip. The end window was then polished by hand to obtain a clean optical surface. To provide strain relief between the tubing (FT900Y, Thorlabs, Newton, New Jersey, United States) of the single-mode fiber and the thin needle, both were glued to a 30-mm-long stainless-steel tube (Unimed S.A., Lausanne, Switzerland) with a diameter of 1.4 mm. The inner needle probe was placed in an outer catheter made from a transparent and biocompatible plastic tube (Fikst Product Development, Woburn, Massachusetts, United States) with an outer diameter of 900  μm, a wall thickness of 200  μm, and a length of 80 mm [Fig. 1(b)]. At the distal end of the catheter, a sharp lancet tip made of stainless steel enables tissue penetration. The proximal end is equipped with a Luer–Lock connection. Figure 1(c) shows the inner OCT probe connected to an ultrasonic piezo translation stage (M2345.663.24, Physik Instrumente, Karlsruhe, Germany) with 20-mm stroke and 0.1-μm resolution as well as a stationary base plate with Luer lock adapter to connect the outer catheter. To reach high imaging speed, the movement of the translation stage was driven by a sinusoidal wave. The OCT was triggered by the stage controller (C-867, Physik Instrumente, Karlsruhe, Germany) at equidistant positions during the movement of the piezo. The advantage of this implementation is that the B-scans needed no postprocessing to achieve equidistant A-scans.

The needle probe was adapted to a spectral domain OCT system (TELESTO II, Thorlabs, Newton, New Jersey, United States) that has a center wavelength of 1300 nm, an axial resolution of 5.5  μm in air, and a depth range of 3.5 mm [Fig. 1(d)]. The interferometer used a 50:50 broadband fiber coupler (TW1300R5F2, Thorlabs, Newton, New Jersey, United States), which was connected to the OCT system. The reference arm comprised a fixed focus fiber collimator (F2800APC-C, Thorlabs, Newton, New Jersey, United States), an iris diaphragm (SM05D5D, Thorlabs, Newton, New Jersey, United States) for attenuation of the reference intensity, and a retroreflector (PS974M-C, Thorlabs, Newton, New Jersey, United States). The traversing needle probe was connected to the second arm of the fiber coupler. Data processing, including numerical dispersion correction and display of the OCT data, was performed in real time using ThorImage software (Version 5.1, Thorlabs, Newton, New Jersey, United States) during the measurement. To achieve a bandwidth-limited depth resolution, numerical fifth-order dispersion compensation was used. Correction coefficients were determined by optimization^19^ of resolution in a prerecorded representative data set.

#### 2.1.1 OCT measurements

For measurements, tissue specimens were placed in thermal contact below a Peltier element [Fig. 1(e)]. A vertical setup holds the translation stage with the traversing needle probe and the cooling/heating device, which consists of a Peltier element with a 2-mm central bore. Temperature was regulated by a thermoelectric controller (5240 - TECSource, Arroyo Instruments, San Luis Obispo, California, United States). The other side of the Peltier element was water-cooled (Oasis 160, Solid State Cooling System, Wappingers Falls, New York, United States). Porcine skin samples were obtained from a local slaughterhouse and stored in the refrigerator at ∼6°C until use. During preparation and handling, porcine fat was warmed to a temperature of ∼10°C. After the tissue was brought into contact with the Peltier element, the needle probe was inserted from above into the tissue. A thermocouple to measure the tissue temperature was placed in the field of view of the OCT. Once the cooled tissue samples and the measurement probe were placed, the Peltier element continuously heated the tissue to 40°C. During the heating, OCT B-scans were continuously measured at a 2-Hz acquisition rate.

### 2.2 Optical Performance

**Fig. 2:**
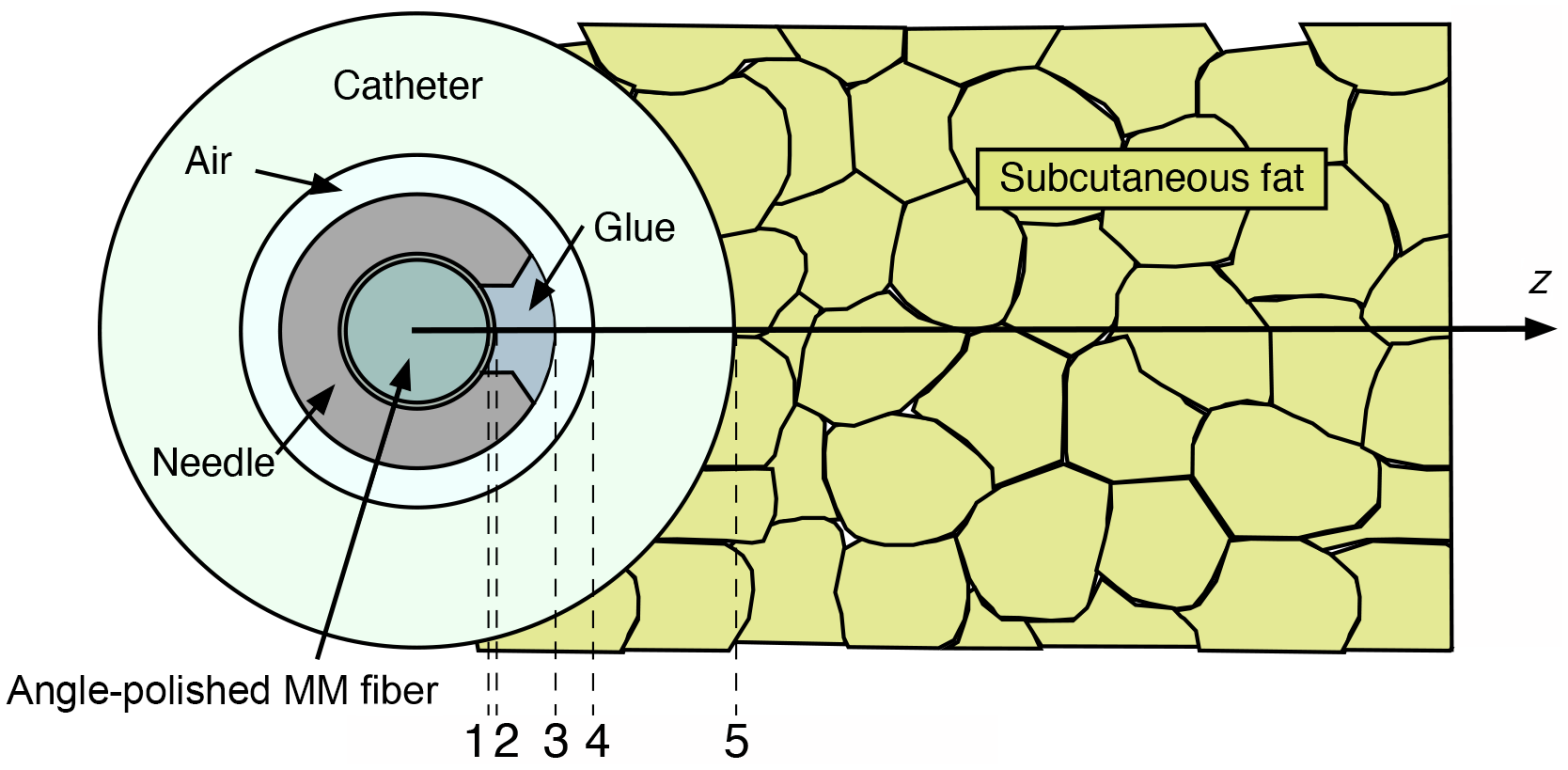
Cross-sectional view of the traversing needle probe at the beam exit window. Between the fiber-based optic and the fatty tissue, the light beam passes through five different cylindrical surfaces (1 to 5), which act as positive or negative cylindrical lenses, depending on the refractive indices of the respective media.

An inherent drawback of small, side-viewing needle probes is the presence of astigmatic aberrations caused by the cylindrical shape of the catheter window (Fig. 2). The amount of astigmatism can be reduced by carefully choosing optical components such that the astigmatisms inherent to the optical components cancel each other out. The CTNP contains a total of five cylindrical interfaces that act as either positive or negative cylindrical lenses, depending on their refractive indices (Fig. 2 and Table 1).

**Table 1:** Cylindrical surfaces in the beam path.

Surface		$n_{i1}$	$n_{i2}$	$r_{i}$ (μm)	$z_{i}$ (μm)
1	Core/cladding	1.447	1.43	100	—
2	Cladding/UV glue	1.43	1.55	110	10
3	UV glue/air	1.55	1	195	85
4	Air/catheter 1, 2, 3	1	1.34, 1.489, 1.592	250	55
5	Catheter 1, 2, 3/tissue	1.34, 1.489, 1.592	1.47^20^	450	200

The first and innermost cylindrical surface is the interface between the core and the cladding of the multimode fiber. The second interface is the boundary between the cladding and the UV adhesive. The third interface is the cylindrical interface between the UV adhesive and the air gap. The fourth interface is the air gap between the needle and the catheter. Although the previous materials cannot be changed, the catheter can be made of different materials with the purpose of minimizing the astigmatic error. The fifth and outermost cylindrical surface is the interface between the catheter and the subcutaneous fat. Beam propagation was simulated for three different catheter materials. The corresponding refractive indices $n_{i1}$ and $n_{i2}$, the radii $r_{i}$, and the distance $z_{i}$ between the cylindrical surfaces are listed in Table 1.

Profiles of the OCT beam were calculated for three different catheter materials commonly used in catheter construction: (1) fluorinated ethylene propylene (FEP) with a refractive index of n=1.34, (2) polyethylene (PE) with n=1.489, and (3) polyethylene terephthalate (PET) with n=1.592 using the split-step beam propagation method (SS-BPM). Publicly available data^21^^,^^22^ for the typical refractive indices of the materials were used, and Sellmeier dispersion fitting was applied to extend the data into the NIR range, where necessary. The refractive power of the cylindrical elements was modeled based on their impact on the phase of the propagated beam.^23^ The influence of the interfaces on the phase was approximated by the complex amplitude transmission function, T, which is given by

$$T=a(x,y)e^{j\phi (x,y))},$$ (1)

where a(x,y) is the aperture function and ϕ(x,y) is the phase change. The coordinates x and y define the plane perpendicular to beam propagation direction z. The phase change was derived from the focal length of the cylindrical surface, f, which is given by

$$f=\frac{r_{i}}{n_{i1}-n_{i2}},$$ (2)

where $r_{i}$ is the radius of curvature of the refractive surface, $n_{i1}$ is the refractive index of the medium in front of the cylindrical surface, and $n_{i2}$ the refractive index of the medium outside of the cylindrical surface. The quadratic phase function, ϕ(x,y), can thus be determined by

$$\phi (x,y)=\frac{k}{f}\sqrt{1+f^{2}y^{2}},$$ (3)

where k is the central wavenumber of the OCT system.

## 3 Results

### 3.1 Astigmatism Compensation

**Fig. 3:**
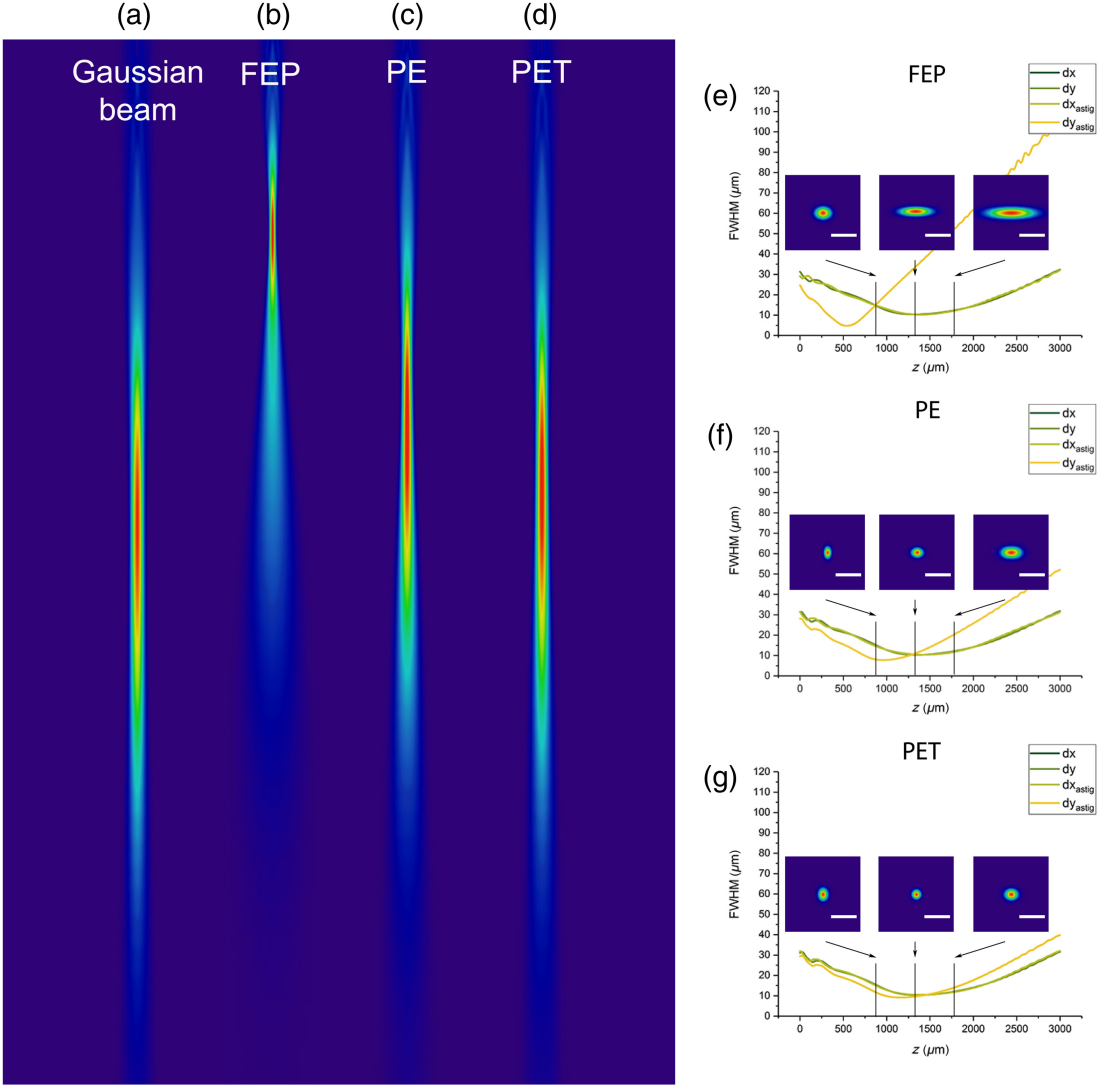
Simulation of beam profiles for three different catheter materials. (a)–(d) False color image of irradiance along the beam propagation (xz plane) for a Gaussian beam (a) and probe with FEP, n=1.34 (b), PE, n=1.489 (c), and PET, n=1.592 (d). (e)–(g) Beam diameter and beam profile along the beam propagation show the astigmatic errors. Each diagram shows the FWHM of the simulated Gaussian beam in the x and y directions, as well as the cross-section through the beam profiles in the x and y plane with the different catheter materials. These curves and the lateral beam profile clearly show the astigmatic aberrations for FEP and PE. Minimal astigmatism is observed for PET. Z axis is the direction of the catheter (Scalebar 50  μm).

Astigmatism was reduced by choosing the best-suited catheter material. For each of the three types of plastics tested, i.e., FEP, PE, and PET, the calculated beam profile of the OCT beam was evaluated in comparison to an ideal Gaussian beam. The different indices of refraction not only determine the focus position [Figs. 3(a)–3(d)] but also influence astigmatism [Figs. 3(e)–3(g)]. Nearly astigmatism-free profile results for the PET material are shown in Fig. 3(g).

For quantitative assessment of the beam quality, the full-width half-maximum (FWHM) along the beam profile was determined in the x- and y-direction from the simulated beam profiles at different distances from the probe. For both directions, the position of the beam waists ($z_{dx}$ and $z_{dy}$), the diameters (dx and dy) at $z_{dx}$, and the ellipticity (dx/dy) were calculated. The results are summarized in Table 2. Azimuthal and longitudinal foci are shifted by 858  μm for FEP, 474  μm for PE, and 225  μm for PET. The spot sizes at $z_{dx}$ in the longitudinal direction are ∼10.4  μm. In the orthogonal direction, the focus diameter for FEP was 36.4  μm, resulting in an ellipticity of 3.6. For PE, the beam diameter in the azimuthal direction is reduced to 13  μm, the ellipticity to 1.3. When using PET as the catheter material, the focus diameter at $z_{dx}$ differed only by 0.4  μm, resulting in an almost round spot with a deviation of 4% from an ideal circle. In conclusion, the use of PET with an index of refraction of 1.592 as the catheter material cancels out the astigmatism almost entirely.

**Table 2:** Beam characterization for three different catheter materials.

Catheter material		FEP	PE	PET
Working distance	$z_{dx}$ (μm)	540	967	1155
	$z_{dy}$ (μm)	1398	1441	1384
Spot size (FWHM) at $z_{dx}$	dx (μm)	10.2	10.4	10.4
	dy (μm)	36.4	13.0	10.0
Ellipticity at $z_{dx}$	dy/dx	3.6	1.3	1.0

### 3.2 Imaging of Porcine Skin

**Fig. 4:**
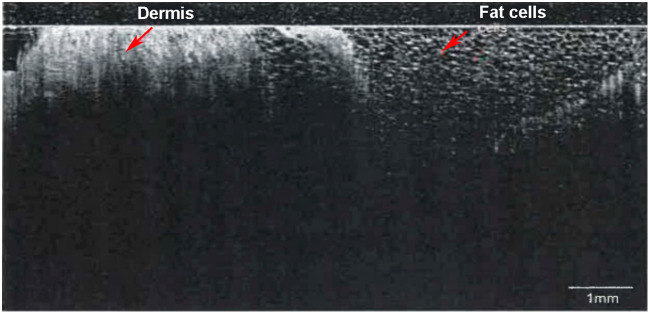
Visualization of a 10-mm-long continuous OCT scan of the dermis and subcutaneous fat by inserting the traversing needle probe perpendicularly to the surface into the skin. The high resolution of the optimized focusing optics enables imaging of individual fat cells.

The image quality of the CTNP was validated by inserting the probe perpendicular to the epidermis in an *ex vivo* porcine skin sample (Fig. 4). Compared with conventional OCT imaging of skin, the B-scan of the needle probe looks unfamiliar because of the viewing direction parallel to the tissue surface. The only limitation of the imaging depth is the insertion depth, and the image quality is depth-independent. In this work, CTNP was able to visualize the skin at a depth of up to 10 mm covering an area from the skin surface to the subcutaneous tissue. Greater scan depth can be achieved with a longer probe and longer stroke of the translation stage. The probe allows for continuous monitoring of all tissue layers with equal quality. In deeper layers of tissue, individual adipocytes can be identified by their dark inner structure and bright cell border (right red arrow).

### 3.3 Monitoring of Temperature-Induced Phase Changes in Fatty Tissue

**Fig. 5:**
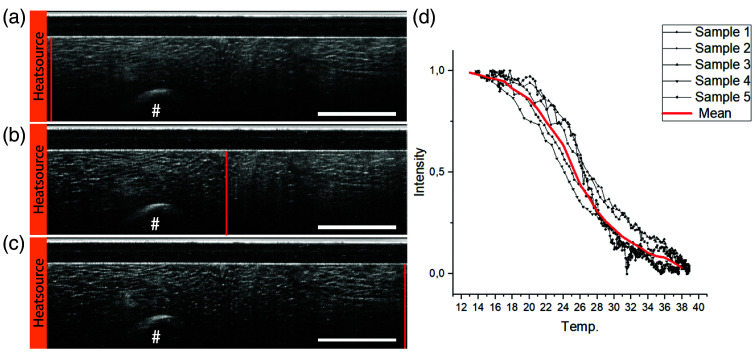
Temperature-induced phase change of porcine subcutaneous fat can be visualized and quantified by OCT imaging with a catheter traversing needle probe (CNTP). For local temperature measurements, a thermocouple (#) was placed in the FOV of the OCT. (a) At the beginning of the heating process at 12°C, high scattering is visible in all fat cells. (b) Heating the (left side of the B-scan) resulted in a gradual decrease of mean scattering from left to right. After 100 s of heating, about half of the scan, up to the vertical red line, shows significantly reduced mean scattering leading to an increased visibility of the cellular texture. (c) After 300 s of heating, scattering is significantly reduced in the entire FOV. (d) Change in the intensity of the OCT signal at the position of the thermocouple in relation to the measured temperature in five different samples. The length of the scalebar is 1 mm.

Temperature-related scattering changes of subcutaneous fatty tissue were measured in five porcine skin samples. An increase in the tissue temperature from 12°C to 35°C resulted in a significant change in the scattering properties (Fig. 5). The heating process was measured with a thermocouple, which was placed in the OCT’s field of view. With increasing temperatures, tissue scattering changed, and individual fat cells became visible. At temperatures below 20°C, fat crystallizes, and subcutaneous tissue shows a higher OCT signal [Fig. 5(a)]. As the heat source was placed on the left side of the B-scan, scattering changed from left to right [Fig. 5(b)]. Five minutes into the experiment, a significant reduction in average scattering can be observed within the entire subcutaneous fat [Fig. 5(c)]. As the cell border of individual adipocytes is clearly delineated by a thin bright cell border, the size of individual adipocytes can be visualized and measured.

To quantify the temperature-induced phase change of subcutaneous fat, signal intensities of several pixels in the vicinity of the thermocouple were averaged and then normalized between 1 (crystallized fat) at the start and 0 (liquid fat) at the end of imaging [Fig. 5(d)]. The normalized averaged signal decreased continuously with temperature until it reached an intensity minimum at 38°C. The curve shape is typical for phase changes^9^ with an inflection point around 26°C for all five samples.

## 4 Discussion and Conclusion

OCT has improved tremendously in speed and resolution since its invention in 1990, but the imaging depth is still limited at around 2 mm in scattering biological tissues. The traversing needle probe has proven to be an effective way to overcome this limitation as it is possible to visualize structures far below the dermis. Here, this concept was extended to a larger FOV using a transparent plastic catheter instead of a second-larger stainless-steel needle with a window on the side. For imaging the phase change of subcutaneous adipocytes with our newly designed catheter traversing needle probe (CTNP), we chose a depth of up to 10 mm, although the imaging depth is only limited by the length of the plastic sheathing and the stroke length of the piezo stage which can be several centimeters long. The transparent catheter prevents direct contact between the needle probe and the surrounding tissue and ensures good and consistent image quality. Although the catheter is intended for single use, the fiber with compact distal optics can be used multiple times, which is a significant improvement compared with wasteful single-use systems.

One disadvantage of this concept is the necessity of a long working distance of the focusing optics because the catheter wall thickness of 200  μm. Thinner plastic catheters do not provide sufficient mechanical stability to pierce the skin. Therefore, the total diameter of the probe was 900  μm. A general problem with the small diameter of OCT needle probes is that their focusing optics have a short working distance. Here, we were able to design an optical setup, which shifts the focus from typically 600 to 1400  μm. The numerical aperture and a resolution of 0.025 and 10  μm, respectively, are maintained using a low NA GRIN fiber, which is matched to the used single-mode fiber. Thus, an increase in working distance by a factor of 2.3 was achieved at the same resolution and overall diameter of the probes previously reported.^16^ Long working distance is a precondition for image quality and depth comparable with those of nonendoscopic OCT systems. Another common optical problem of needle probes is astigmatic aberrations, which are caused by the cylindrical shape of the focusing optics. To get close to a limited-diffraction resolution in tissue, considerable efforts were made by introducing additional reflective and diffractive elements,^24^ printed free-form optical components,^25^ and metalenses.^26^ In this work, we focused on combining materials with different refractive indices to minimize astigmatism. Using wave propagation–based simulations was not only valuable for achieving rapid design iterations but also gave a deeper understanding of material compositions and also allowed us to make the best use of a combination of different materials. However, simulations have limitations in predicting the exact beam profile, especially as refractive indices of plastics can vary from batch to batch, and many manufacturers are unwilling to provide precise specifications. Furthermore, the optical properties of tissue may undergo significant changes at different temperatures.^27^ Despite these limitations, the simulations were instrumental in identifying the optimal catheter material for the CTNP.

The monolithic design of the probe is another important advantage because no microoptical elements or special adjustments are necessary. Using a glass processor, where cleaving and splicing are done under camera control, and without the need for a transfer of the fiber, a repeatability of 5  μm in cleave length was achieved. This corresponds to a maximum variation in working distance and spot size of just 2.5%. In general, an efficient and reproducible production of large quantities of probes is possible.

Using excised porcine skin, we were able to show that the CTNP can reproducibly measure temperature-dependent phase changes of subcutaneous fat with a high spatial resolution. Previous research has shown a significant change in the refractive index of lipid pools at various temperatures.^27^ However, we have not observed a substantial impact on image quality. In addition, this effect is less relevant to our current application, but it needs to be taken into account in probe design for other applications where structural information is more relevant. The measured melting temperature of 26°C falls well within a literature-confirmed range of 21°C to 36°C for porcine fat. The actual value depends on diet, weight, and body region.^28^ The CTNP could be used to elucidate the underlying mechanisms of cryolipolysis and foster further research in this area. The current CoolSculpting treatment protocol is identical for all individuals receiving treatment, with variations in treatment timing based only on the area being treated.^29^ However, it has been shown that the melting point of fat has a wide temperature range based on factors such as composition (saturated versus unsaturated fat ratio) and body region.^30^ Therefore, we are proposing that a qualitative assessment of each individual’s fat depot-specific crystallization temperature could increase treatment efficacy. Given that fat crystallization, or not fat temperature, is likely the relevant factor, simply inserting thermocouples will not lead to optimal treatment control. However, being able to analyze fat in real time using an OCT needle probe could define fat crystallization as a dosimetry parameter. Due to the invasive nature of this needle OCT probe, it is likely not to become part of the typical clinical selective cryolipolysis procedure. However, this imaging probe can help to optimize the dosimetry and improve the procedure in a research setting.

In addition to being able to visualize temperature-related changes in subcutaneous fat, we envision the traversing OCT needle probe to also be used for other dermatologic applications, such as real-time morphological analysis to guide filler injections,^31^ or analyzing the composition of cellulite and various dermatologic anomalies.^32^^,^^33^

Although this study focused on increasing the depth range and image quality of the traversing OCT needle, further research will look into creating smaller needles using smaller fibers and catheters for less invasive applications. By adding a rotation table, our probe would be able to acquire 3D images, which would allow an even more robust visualization of dermal, epidermal, and adipose tissue layers and allow clusters of nonreactive fat cells to be identified more easily. However, the speed of the OCT system would have to be increased using a swept source OCT system instead of a high-speed spectrometer-based OCT system.

In summary, we presented an optical design for a traversing OCT needle probe that is easy to manufacture, is reusable in several subjects, causes minimal beam distortions, and is able to visualize several centimeters of tissue. Furthermore, our study has reproducibly demonstrated the utility of an OCT needle probe for the visualization of temperature-induced phase changes in subcutaneous fatty tissue. The presented needle probe is a new and useful imaging tool not only to study the process of selective cryolipolysis but also to visualize and quantify fatty tissue, which without the presented needle probe is not accessible to OCT imaging.

## Data Availability

The data supporting the findings of this paper are not publicly available but may be obtained from the authors upon reasonable request.
